# Primary Bone Tumors of the Spine—Proposal for Treatment Based on a Single Centre Experience

**DOI:** 10.3390/diagnostics12092264

**Published:** 2022-09-19

**Authors:** Nicole Lange, Ann-Kathrin Jörger, Yu-Mi Ryang, Friederike Liesche-Starnecker, Jens Gempt, Bernhard Meyer

**Affiliations:** 1 Department of Neurosurgery, Klinikum Rechts der Isar, Technical University, 81675 Munich, Germany; 2 Department of Neurosurgery, Helios Klinikum Berlin-Buch, 13125 Berlin, Germany; 3 Department of Neuropathology, Institute of Pathology, Technical University, 81675 Munich, Germany

**Keywords:** primary bone tumor, outcomes, treatment strategy

## Abstract

This study reports a large single-center series of primary bone tumors of the spine (PBTs). We aimed to review the concepts for management, as this kind of tumor represents a very rare entity, and also propose a new treatment algorithm. Retrospective analysis revealed 92 patients receiving surgery for PBTs from 2007 to 2019 at our center. They were analyzed based on surgical management and the course of the disease. A total of 145 surgical procedures were performed (50 cervical, 46 thoracic, 28 lumbar, and 21 sacral). Complete tumor resection was achieved in 65%, of which 22% showed tumor recurrence during follow-up (mean time to recurrence 334 days). The five-year mortality rate was significantly lower after complete resection (3% versus 25% after subtotal resection). Most of the patients improved in their symptoms through surgery. Regarding the tumor entity, the most common PBTs were vertebral hemangiomas (20%), osteoid osteomas (15%), and chordomas (16%). The Enneking graduation system showed a good correlation with the risk of recurrence and mortality. Complete resection in PBTs increased survival rates and remains the method of choice. Thus, quality of life—especially with a higher extent of resection—should be considered.

## 1. Introduction

Primary bone tumors (PBTs) of the spine represent a heterogeneous group of both malignant and benign tumors that are less common than metastases or multiple myeloma. They comprise about only 0.2% of all newly diagnosed tumors every year (5% within the group of bone tumors) [1]. Despite their heterogeneity, for a PBT, an accurate differential diagnosis can be established by taking into account the patient’s age, the radiological pattern, and the topography of the lesions [2]. During the first decade of life, >90% of spinal tumors are benign, about 50% in the fourth decade, and less than 10% in the seventh decade [3]. While some, mostly benign, PBTs are mainly located in the anterior vertebral body (e.g., eosinophilic granulomas, vertebral hemangiomas, and giant cell tumors), others are predominantly seen in the posterior elements (e.g., aneurysmatic bone cysts, osteoid osteomas, osteoblastomas, and osteochondromas) [4,5,6]. Chordomas, chondrosarcomas, Ewing’s sarcomas, and osteosarcomas are the most frequent malignant PBTs. Some semi-malignant lesions, such as giant cell tumors, benign aneurysmatic bone cysts, and osteoblastomas can have aggressive behaviors and reoccur if not treated properly [7,8]. Complete surgical resection followed by adjuvant treatment remains the gold standard [9]. Concerning the outcome of malignant PBTs, recurrence and five-year-mortality rates are high (up to 48% and 58%, respectively) [10,11,12]. Because this is a rare disease, mortality rates and recurrence data are only based on case series or reviews.

In this study, we report a single-center experience of 92 consecutive cases of symptomatic PBTs treated surgically. The objective was to report our experience, with a focus on the morbidity of en-bloc resections and the decision-making procedure; thereby challenging the status quo on symptomatic benign and malignant PBTs.

## 2. Materials and Methods

We searched the department database for all patients who underwent spinal surgery for symptomatic tumors of the spine in a 12-year period from January 2007 to August 2019, followed by a pathological diagnosis of primary bone tumor. In 3% of cases, tumors were incidental findings on MRI scans, which were resected because they were large or causing instability. The present study was approved by the local ethics committee (local ethics committee of the Technical University Munich) and performed in accordance with the ethical standards established by the 1964 Declaration of Helsinki and its later amendments [13] (Clinical Trial Registration Number: 205/18S).

Patient and disease characteristics were recorded, including age, affected vertebrae, date and type of surgical procedures, neurological symptoms before and after surgery, preoperative treatments, such as embolization, as well as postoperative adjuvant radiotherapy. Since the study was a retrospective analysis, patients’ informed consent was waived by local ethics committee of the Technical University of Munich. The study has been registered on DRKS (trial registration number: DRKS00023989).

Radiographically, all lesions were characterized according to their SINS score [14], the Enneking-classification system for benign and malignant tumors [15], and the Weinstein–Boriani–Biagini classification [1]. Within the Enneking classification system, benign lesions were classified as follows: S1 = lesions are inactive asymptomatic slowly- or non-growing, with a true capsule; S2 = lesions are mildly symptomatic slowly growing with enlarged tumor outlines, and show a low recurrence rate; and S3 = lesions are rapidly growing with breached or absent tumor capsule and invasion of neighboring structures, and show a high recurrence rate. Malignant tumors were classified based on their grade (1: low versus 2: high), local extension (A: intra- vs. B: extra-compartmental), and the presence of metastases (M) [16,17]. The largest diameter in sagittal MRI was recorded.

Regarding outcomes, calculation was done using the Karnofsky performance status scale (KPS) at the time of admission and during the follow-up period (after 3 and 6 months, and again 1, 3, and 5 years after surgery, depending on the initial surgery date). Data were taken from our database. Mortality and complication rates, as well as progression-free survival, were assessed.

As this study was a retrospective analysis, patients’ informed consent was not necessary.

Statistical analyses, including descriptive data analyses, were performed using IBM SPSS Statistics version 22.0 (IBM Corporation, New York, NY, USA). Associations between nominal variables were analyzed using chi-square tests, and continuous variables were analyzed using binary logistic regression analysis. For all analyses, a difference with an error probability of less than 0.05 was considered statistically significant. Descriptive statistics for demographic variables were generated with means and SDs, or medians with interquartile ranges, as appropriate.

## 3. Results

### 3.1. Patient Population

Between January 2007 and August 2019, we identified 92 patients (59 male, 33 female) who received surgery for a PBT at our center. During this period, the center saw exactly 1000 patients undergo surgery for spinal metastases. This gave PBT surgery a percentage of 8.4% of all spinal bone tumor surgeries. The median age was 46 (ranging from 7 to 80). Interestingly, the mean age within the groups of benign and malignant lesions was 40 and 47, respectively, and did not differ significantly. In terms of localization, 30% were at the cervical spine, 34% at the thoracic spine, 21% at the lumbar spine, and 15% at the sacrum.

In total, 97% of the lesions were symptomatic, with permanent and movement-sensitive local pain. In addition, 36% of the patients showed neurological deficits, such as pareses (19%), dysesthesia (25%), and myelopathy (13%). A total of 3% of tumors were incidental findings on MRI scans. Those were either chordomas or fibrosarcomas. There was no correlation of neurological compromises with the Weinstein–Boriani classification (*p* = 0.18) or the Enneking classification (*p* = 0.51), nor the SINS score (*p* = 0.74). Referring to co-morbidities, 17% of the patients showed malignant tumors of other origin, which were treated by surgery, followed by radio- and chemotherapy.

### 3.2. Classification of the Lesions

**Figure 1:**
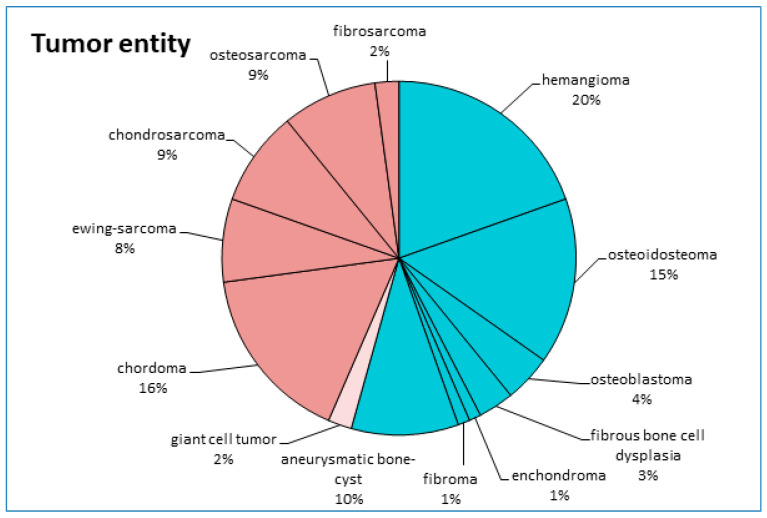
Tumor entities. Exact differentiation of tumor entities according to histopathological results. Total number of patients: 92. Groups divided into benign lesions (blue), semi-malignant lesions (beige), and malignant tumors (red).

For tumor entities, 54% were benign lesions, 2% were semi-malignant, and 44% were malignant tumors. The exact differentiation of tumor entities according to histopathological subgroups is presented in Figure 1. The most common PBTs were vertebral hemangiomas (20%), osteoid osteomas (15%), and chordomas (16%).

In terms of radiographic classification, the mean SINS score was 8 (ranging from 3 to 14). Concerning the Enneking scoring system for benign spinal lesions, 40% were S1 staged lesions, 26% were S2, and 34% were classified as S3. Within the group of malignant and semi-malignant tumors, 10% were Enneking 1A lesions, 24% were 1B, and 14% were 2A. Half the malignant tumors were classified as Enneking 2B. Nine of these patients (45% of Enneking 2B) showed systemic metastases at the time of presentation.

The mean tumor size, measured as the largest diameter in sagittal T2 MRI sequences, was 27 mm (ranging from 4 to 98). One lesion was measured using myelo-CT, as the patient’s pacemaker was not suitable for MRI. Meanwhile, 45% of the patients showed extraosseous intraspinal (either epi- or intradural) tumor, characterized by Weinstein–Boriani–Biagini D or E.

### 3.3. Treatment

**Figure 2:**
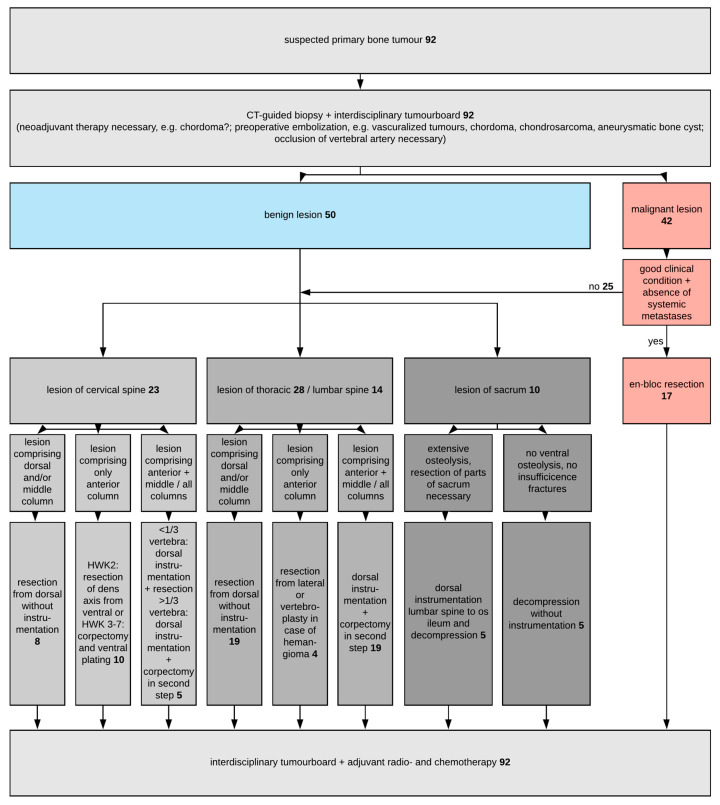
Flow-chart of treatment in case of primary bone tumors.

Figure 2 shows a flow-chart of the proposed treatment algorithm based on our single-center experience.

In the case of a suspected primary bone tumor, a CT guided biopsy was conducted, followed by an interdisciplinary tumor board. In the case of histopathologically proven chordomas, neoadjuvant radiotherapy is recommended. Preoperative embolization of the tumor feeding vessels should be considered for vascularized tumors (chordomas, chondrosarcomas, aneurysmatic bone cysts, and giant cell dysplasias) or in cases where occlusion of the vertebral artery is necessary. In our patient collective, 13% of the patients underwent preoperative embolization of the tumor. Their cases were all benign S3 lesions, or malignant stage B tumors (with tissue reactions surrounding the vertebra).

In the case of malignant lesions, en-bloc resection has to be considered. Due to its increased morbidity, en-bloc resection is recommended in the case of a good clinical condition of the patient, without any systemic metastases or other malignant diseases. Aggressive en-bloc resections were conducted in 17 cases. They were all sarcomas and chordomas. Nevertheless, eight of them suffered recurrent tumors during the follow-up time.

Four procedures for thoracic tumors (8% of all thoracic surgeries) were interdisciplinary surgeries with thoracic surgeons, continuing with partial lung resections. For cervical lesions, total resection of the tumor is conducted via dorsal resection, with or without stabilization, corpectomy and ventral plating or dorsal stabilization, followed by corpectomy and ventral plating in a second step, depending on the localization and extent of the tumor. Concerning the thoracic and lumbar spine, lesions are resected dorsally or laterally without instrumentation, or via dorsal stabilization followed by corpectomy in a second step. Sacral lesions are either resected from the dorsal or additionally stabilized when osteolysis involves large parts of the sacral bone or resection results in instability of the sacroiliac joint.

In all cases, adjuvant radio- and chemotherapy and/or follow up MRI is recommended after surgery, depending on the histopathological findings. We conducted 1.6 surgeries per patient (range 1 to 4), followed by radiotherapy in 45% (protons in 3%) and chemotherapy in 17%. Surgeries were tumor resections and decompressions in 54% and instrumentations in 51% (48% of those with combined dorsoventral approaches, including spondylectomy and vertebral body replacement either with cages or with autologous fibular implants [2 patients]). The mean SINS score in the group of instrumentation was 9.

In 65%, complete resections were achieved. There was one case of perioperative death. The patient suffered severe pneumonia following partial lung resection for thoracic chondrosarcoma. He died during his hospital stay. Complications occurred in 10%, leading to revision surgeries in eight cases (implant failure in five cases, tumor remnant in two, and CSF leakage in one).

### 3.4. Outcome

**Figure 3:**
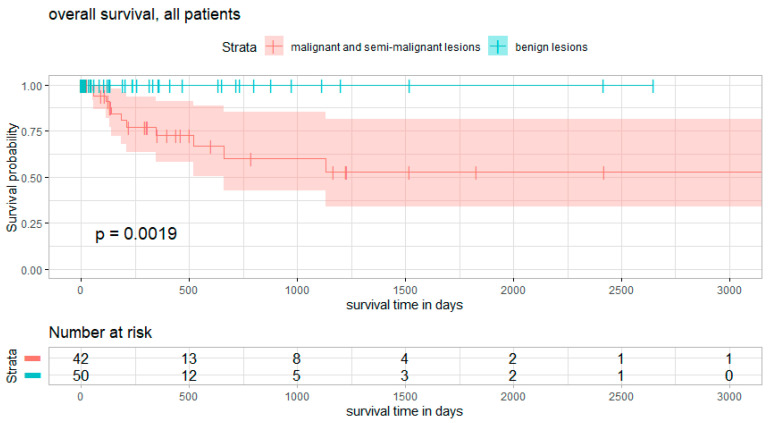
Overall survival. Survival curves for overall survival, subdivided into subgroups of benign lesions (blue) and malignant/semi-malignant lesions (red). Curve comparison shows significant differences between curves. Log rank test of benign vs. malignant/semi-malignant lesions: X^2^ = 9.63, *p* = 0.00019.

**Figure 4:**
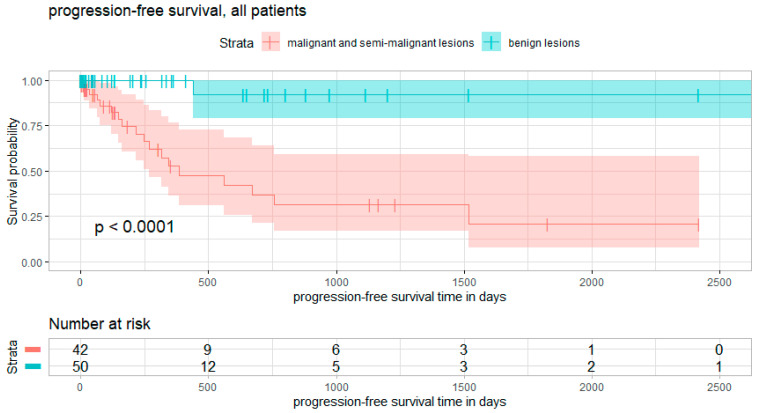
Progression-free survival. Survival curves for progression-free survival, subdivided into the subgroups of benign lesions (blue) and malignant/semi-malignant lesions (red). Curve comparison shows significant differences between curves. Log rank test of benign vs. malignant/semi-malignant lesions: X^2^ = 18, *p* < 0.0001.

Overall survival- and progression-free survival curves are presented in Figure 3 and Figure 4, respectively.

The mean follow-up time was 438 days (ranging from 10 to 3172 days). During the follow-up period, overall mortality was 12%. No death occurred in the case of benign lesions (log rank test benign vs. malignant/semi-malignant lesions: X^2^ = 9.63, *p* = 0.00019). Median survival was not reached during the follow-up period.

In all, 45% of patients dying during follow-up suffered metastatic tumor at the time of presentation. Mortality after complete resection and after subtotal resection was 3% and 28%, respectively. A risk factor analysis is shown in Table 1, Table 2 and Table 3, revealing sarcoma (*p* = 0.000059), malignant tumors (*p* = 0.000054), Enneking stage 2 lesions (*p* = 0.000004), and subtotal resections (*p* = 0.0049) as significant risk factors for increased mortality. Binary logistic regression analysis showed age as a significant factor for increased mortality (*p* = 0.04). SINS score, KPS at admission and discharge, tumor size, and lesions classified as Weinstein–Boriani D/E had no positive correlation. A subgroup analysis of malignant lesions confirmed subtotal resection as an independent risk factor for increased mortality (*p* = 0.02), while en-bloc resection did not show significantly better survival rates.

A total of 21% of patients suffered a recurrence of the tumor during the follow-up period. The mean time to recurrence was 334 days (ranging from 12 to 1519 days). Within the group of benign tumors, only one patient suffered recurrence after 441 days. This was a 78-year-old female with fibrous dysplasia of the cervical spine, undergoing surgical decompression. One of the two giant cell tumors reoccurred 118 days after instrumentation and radiologically proven complete resection. Log-rank test benign versus malignant/semi-malignant lesions: X^2^ = 18, *p* < 0.0001.

Analogously, our analysis showed that patients with malignant lesions had a 27-times higher risk of recurrence (*p* = 0.000001). For Enneking stage 2 lesions, the risk of hazard was six times higher (*p* = 0.000025). Epidural tumors (classified as Weinstein-Boriani D or E) did not show a significant higher risk of tumor recurrence. Subtotal resection, primary surgical procedure and age were also not significant risk factors for higher recurrence rates. None of those factors were significant in binary logistic regression analysis.

Regarding clinical outcomes, 73% of patients showed clinical improvement directly after surgery; 25% did not show any immediate changes, while three patients suffered a worsening of neurological functions. These three cases were as follows: a case of osteosarcoma of the cervical and thoracic spine showing new paresis postoperatively; a case of sacral chordoma sensing worsening in bowel control; and a case of an aneurysmatic bone cyst that caused an increase in pain after surgery.

The mean KPI was 80% at admission and during the first year of follow-up. Thereafter, it decreased to 70% and worsened up to a mean KPI of 50% after the fifth year of follow-up, due to the systemic progression of underlying malignancies.

**Figure 5:**
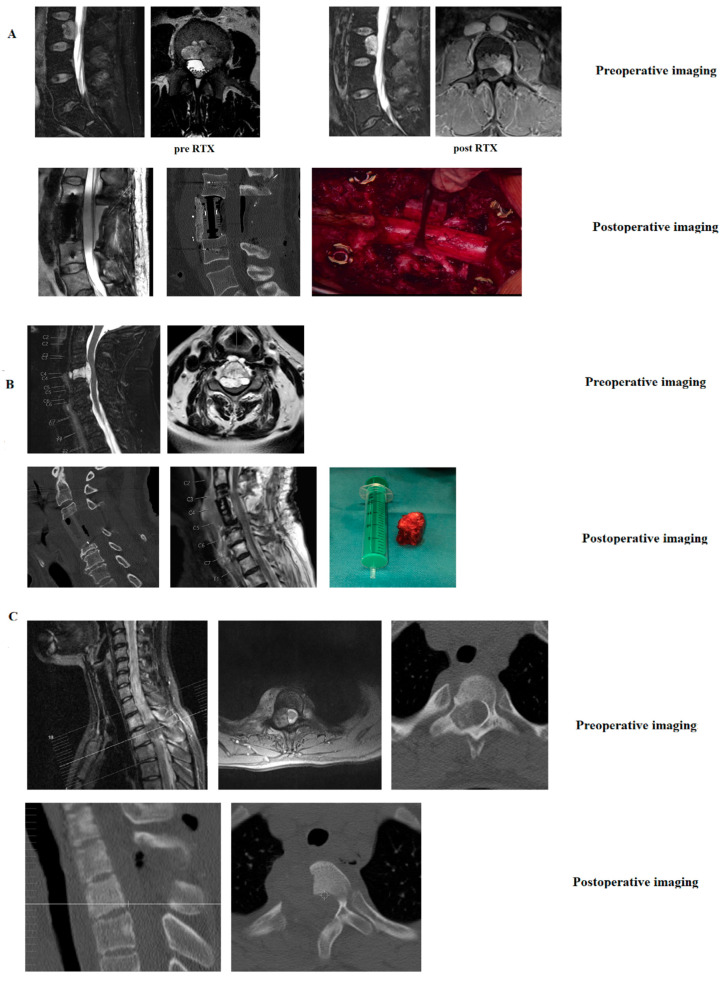
(**A**–**C**) Case descriptions.

Case descriptions (Figure 5).

#### 3.4.1. Case 1: Chordoma of the Lumbar Spine (Figure 5A)

The patient was a 55-year old female with low back pain and left-sided L5 radiculopathy for 3 months. MRI showed a tumor in LWK 5, comprising all three vertebral columns. CT-guided biopsy showed chordoma. Neoadjuvant radiotherapy was administered. Surgery was conducted as follows: Dorsal stabilization L4-S1 with Carbon fiber screws and rods (Icotec internal fixator), left-sided hemilaminectomy L5, removal of the facet joints in L4/5 and L5/S1, tumor debulking, and inlay of a sterile glove to mark the dural sac and the decompressed nerve roots L4 and L5. Some days later, corpectomy L5, partial corpectomy L4, a continuation of tumor resection via lateral retroperitoneal approach and implantation of a distractible PEEK Cage were conducted. Final postoperative imaging showed complete resection. Postoperatively, the patient was pain-free and a proton beam was applied.

#### 3.4.2. Case 2: Chordoma of the Cervical Spine: (Figure 5B)

The patient was a 38-year-old male with chronic pain in the cervical spine for 6 months. For two days, he experienced difficulties in walking. Examination showed myelopathy, as well as paresis of both arms (elevation and flexion 3/5). MRI showed a tumor of C4 comprising the vertebral body and spinal canal. Preoperative neoadjuvant therapy was not possible because of the developing pareses and neurologic deficits. Therefore, surgery was performed the next day. The surgical procedure comprised corpectomy of C4 and ventral plating. Final postoperative imaging and the removed vertebra are shown in Figure 5.

#### 3.4.3. Case 3: Osteoid Osteoma of C1: (Figure 5C)

The patient was a 23-year-old female presenting with left-sided neck pain, and no radiation. There was pain improvement after treatment with aspirin. Preoperative imaging showed a tumor in C1. The histopathological analysis of the biopsy was not conclusive. Surgery comprised a dorsal approach to the upper cervical spine and resection of the left-sided arch of C1. Postoperative imaging showed complete resection. The patient was pain free. No adjuvant therapy was necessary.

## 4. Discussion

In this recent single-center analysis of 92 patients suffering from PBTs of the spine, excluding plasmocytomas, we clarified the mortality and recurrence rates. By providing a risk-factor analysis and reporting treatment strategies and outcomes, the future decision-making process can be simplified.

In our patient collective, the median age was 46 (ranging from 7 to 80). Interestingly, the mean age within the groups of benign and malignant lesions was 40 and 47, respectively, and did not differ significantly. Other publications describe patient’s age as a good indicator for differential diagnosis of benign versus malignant lesions. This is because >90% of spinal tumors are benign during the first decade of life, about 50% in the fourth decade, and less than 10% in the seventh decade [3]. The most frequent benign lesions were vertebral hemangiomas, osteoidosteomas, and aneurysmatic bone cysts. Chordomas, osteosarcomas, and chondrosarcomas were the most common malignant tumors. This is in agreement with the literature [2,7,18].

Radiographically, all lesions were characterized according to the SINS score [14], the Enneking classification system for benign and malignant tumors [15], and the Weinstein–Boriani–Biagini classification [1]. Interestingly, Even though those classification systems were applied, they did not correlate to any clinical symptoms or neurological state of the patients. Furthermore, the SINS score and the Weinstein–Boriani classification were easily applicable but could not be identified as risk factors for mortality or recurrence. Enneking staged 2 lesions (involving surrounding tissue) showed a significantly higher hazard for mortality and recurrence, as did the histopathologically malignant lesions.

The rate of recurrence in our patient collective stood at 23%, which is far lower than that described in the literature [19,20,21]. The mean time to recurrence was 334 days (in a series of sacral chordoma it was 582 days) [22]. Thus, the patient’s age and the initial surgical procedure seemed not to influence recurrence rates. Overall mortality was 11%. Mortality after complete resection was significantly lower than after subtotal resection (3% vs. 25%). Interestingly, a subgroup analysis of patients with malignant lesions revealed subtotal resection as a risk factor for mortality, whereas en-bloc resection did not influence mortality significantly.

Regarding therapy concepts, we provided a therapy algorithm for PBTs. In the case of benign tumors, complete resections should always be aimed for. The surgical approach depends on exact localization of the tumor, as shown in the algorithm. After surgery, a tumor board is again held to decide on any adjuvant radio- or chemotherapy concepts for each patient, considering postoperative imaging and histopathological results.

An analysis of surgical margins after the resection of PBTs revealed a significant reduction of recurrence (recurrence rate of up to 48%) with respective Enneking-appropriate margins, as well as an increase in survival after aggressive en-bloc resections [12,23,24,25]. In this analysis, subtotal resection was not an independent risk factor for recurrent tumors, but for increased mortality.

Semi-malignant lesions are treated neoadjuvant with radiotherapy or specific antibodies (e.g., denosumab) [26,27,28]. This treatment should be followed by complete tumor resection. However, the morbidity of en-bloc resection is high, such that indications should be critically verified. Some semi-malignant tumors can also be treated via embolization of the tumor-feeding vessels.

Considering the high morbidity of en-bloc resections (comprising complication rates up to 76% [29]), in the case of malignant lesions, they are primarily recommended in patients with a good clinical condition and the absence of systemic metastases, so that complete tumor removal is achievable. Improvements in tumor-related mortality must be balanced against procedure-related morbidity, and local lesion control against the preservation of function. Therefore, patients must be selected carefully and managed by specialized departments in tertiary centers. Patients will then profit the most regarding systemic tumor progression.

Chordomas are treated neoadjuvant with radiotherapy; patients with Ewing-sarcomas receive chemotherapy before surgery. Of course, in cases with progressive neurological deficits, the decision is made for rapid surgery, followed by adjuvant treatment.

It is known that any spreading of tumor cells due to an intial inappropriate surgical procedure already reduces the patient’s prognosis [10]. This underlines the differences in surgical techniques between decompressive surgery in the metastases of the spine and the attempt at complete resection in PBTs. Many PBTs are curable through resection with Enneking-appropriate margins.

Therefore, and especially due to their rarity, patients with PBTs should be treated in spine centers. Apart from complete resections, the importance of genetics is increasing for the development of targeted chemotherapies (e.g., denosumab in giant cell tumors) following tumor resections [26,30,31]. Regarding clinical outcomes, 72% showed clinical improvement directly after surgery, with an initial KPI of 80%. However, with continued follow-up, clinical status seemed to worsen, in line with the higher percentage of progressive disease, emphasizing the severity of this type of uncommon tumor.

The provided treatment algorithm is an expert opinion, based on a long-term experience of a large spine surgery center. Conversely to the outdated thought that en-bloc resection is the state of the art for all primary bone tumors, we advocate a change of view, in order to balance improvements in tumor-related mortality against procedure-related morbidity, and local lesion control against the preservation of function.

## 5. Conclusions

PBTs of the spine are very rare lesions and, as such, are not amenable for RCTs, to provide first-class evidence; to provide reasonable evidence, other forms need to be found. Nevertheless, we suggest questioning the status quo regarding radical resections of high-grade tumors, due to the development of modern neoadjuvant/adjuvant therapies. Although complete resections increase survival rates, postoperative quality of life needs to be considered. Complete resections for benign lesions, however, are considered the method of choice, as they lead to very favorable survival rates.

## Figures and Tables

**Table 1:** Chi-square analysis of binary variables analyzing risk factors for mortality and recurrence for all patients.

	Chi-Square Analysis, Binary Variables	HR	95% CI	X2	*p*
risk factors for mortality, all patients	sarcoma/no sarcoma	12	2.87–50.2	16.13	**0.00006**
	malignant/benign tumour	inf	Inf	16.31	**0.00005**
	Enneking 2A/B/Enneking S1-3; 1A,B	25.62	6.24–105.2	21.29	**0.000004**
	Subtotal resection/marginal resection	6.81	1.89–24.6	7.93	**0.005**
	Weinstein—Boriani D or E/A-C	1.08	0.313–3.75	0.02	0.90
risk factors for recurrence, all patients	malignant/benign tumour	27	10.8–67.6	23.72	**0.000001**
	Enneking 2A/B/Enneking S1-3; 1A,B	6.17	2.21–17.2	17.77	**0.00003**
	Subtotal resection/marginal resection	0.78	0.31–1.99	0.24	0.63
	Decompression/instrumentation	0.68	0.278–1.69	0.64	0.42
	Weinstein—Boriani D or E/A-C	1.49	0.606–3.66	0.75	0.39

**Table 2:** Chi-square analysis of binary variables analyzing risk factors for mortality and recurrence for patients with malignant tumors.

	Chi-Square Analysis, Binary Variables	HR	95% CI	X^2^	*p*
risk factors for mortality, malignant tumors	Enneking 2A/B/Enneking I A/B	5.0	1.43–17.5	2.90	0.09
	Subtotal resection/marginal resection	4.95	1.52–16.2	5.16	**0.02**
	En-bloc resection/other resections	0.36	0.10–1.22	1.85	0.17
	Weinstein–Boriani D or E/A-C	1.09	0.334–3.56	0.02	0.89
risk factors for recurrence, malignant tumors	Enneking 2A/B/Enneking I A/B	1.3	0.488–3.46	0.25	0.62
	Subtotal resection/marginal resection	0.55	0.218–1.39	1.47	0.23
	En-bloc resection/other resections	1.3	0.502–3.37	0.31	0.58
	Weinstein–Boriani D or E/A-C	1.14	0.451–2.87	0.07	0.79

**Table 3:** Binary logistic regression analysis of risk factors for mortality and recurrence for all patients.

	Binary Logistic Regression, Continous Variables	*p*
risk factors for mortality, all patients	Age	**0.04**
	KPS at admission	0.77
	KPS at discharge	0.47
	SINS score	0.81
	Tumour size (sagittal diameter, mm)	0.09
risk factors for recurrence, all patients	Age	0.45
	KPS at admission	0.95
	KPS at discharge	0.76
	SINS score	0.87
	Tumour size (sagittal diameter, mm)	0.08

## Data Availability

Data and additional material can be provided on request.
